# Performance of the Mexican nursing labor market: a repeated cross-sectional study, 2005–2019

**DOI:** 10.1186/s12960-022-00721-4

**Published:** 2022-03-12

**Authors:** Gustavo Nigenda, Edson Serván-Mori, Evelyn Fuentes-Rivera, Patricia Aristizabal, Rosa Amarilis Zárate-Grajales

**Affiliations:** 1 School of Nursing and Obstetrics, National Autonomous University of Mexico, Mexico City, Mexico; 2 Center for Health Systems Research, National Institute of Public Health, Cuernavaca, Morelos Mexico; 3 Center for Demographic, Urban and Environmental Studies, College of Mexico A.C., Mexico City, Mexico; 4 Iztacala Faculty of Higher Studies, National Autonomous University of Mexico, Mexico City, Mexico

**Keywords:** Labor market, Nursing, Performance, Low- and middle-income countries, Mexico

## Abstract

**Background:**

The close link between human resources for health and the performance of health systems calls for a comprehensive study of the labor market. This paper proposes a performance metric for the nursing labor market, measures its magnitude and analyzes its predictors over the last 15 years.

**Design and methods:**

A repeated cross-sectional analysis using data from the quarterly population-based National Survey of Occupation and Employment 2005–2019 (ENOE in Spanish). An aggregate total of 19,311 Mexican nurses (population *N* = 4,816,930) was analyzed. Nursing labor market performance was defined as the level of non-precarious employment of nurses in the health sector. After describing the sociodemographic, labor and contextual characteristics of the nurses surveyed, we identified the key correlates of market performance using repeated cross-sectional multiple logistic regression analysis. We then estimated the adjusted prevalence of market performance according to the survey period and socioeconomic region of residence.

**Results:**

The exogenous indicators analyzed shed light on various aspects of the market structure. Unemployment remained stable at 5% during the period examined, but underemployment rose by 26% and precarious employment, our endogenous indicator, also grew significantly. On the whole, our indicators revealed a notable deterioration in the structure of the nursing labor market; they varied by age and sex as well as between public and private institutions. Although the steepest deterioration occurred in the private sector, we observed an increase in precarious jobs among public institutions formerly protective of employment conditions.

**Conclusions:**

The deterioration of the labor market jeopardizes the ability of nursing professionals to participate in the market as well as to obtain secure jobs once they do enter. The Mexican Health System suffers from a chronic dearth of nurses, reducing its capacity to achieve its core objectives including enhanced coverage and increased effectiveness. Nursing workforce planning requires a context where the conditions in which the market currently operates, and its potential deterioration are considered.

## Introduction

The close link between nursing human resources and the performance of health systems worldwide [1] calls for a comprehensive study of the nursing labor market [2]. Unlike individuals engaged in general labor markets, those who participate in professional labor markets, after accumulating large human capital throughout a long training process, seek employment options that allow them to capitalize on their investment in terms of professional development and salary [3].

Labor markets have undergone significant changes over the last three decades as a consequence of a structural transformation in international economic production [4]. The industrialized countries in Europe, the United States and Canada [5] among others, have adapted their labor markets to the conditions in the post-industrialization period, characterized by the predominance of service production and a shift in the employer–employee relationship resulting in the erosion of labor rights. Two notable aspects of this transition are a rising supply of temporary jobs and unstable employment relations [6], both mirrored by a segmented labor market.

Segmentation is based essentially on an employment structure that includes/excludes prerogatives according to national labor laws and the recommendations of the ILO [7]. Studies have revealed an association between the growing participation of labor in the informal sector and segmentation. They have also documented the negative effects of segmentation on national economies [8], for instance, in the form of reduced tax revenues. This situation prevails across the majority of Latin American countries [9] and suggests a deterioration in labor markets as a whole. Its origins lie in structural macroeconomic changes (globalization), the incorporation of third-party mechanisms such as outsourcing, and a shift in personnel management methods [10].

Increased professionalization and extended training for nurses have posed challenges for structuring the local and global labor markets in this field [11, 12]. One fundamental proposal for meeting these challenges involves the assessment of labor market performance for nursing professionals. Over and beyond exploring market determinants, analyses need to examine its supply–demand relationship in order to ascertain the characteristics of nursing participation and identify variations in performance. Offering sufficient and legally protected employment requires that the design and management of public policy in this area rest on solid evidence as regards the performance of the nursing labor market. This encompasses not only the medium- and long-term production of graduates, but most importantly, the capacity of health systems to deliver services that meet the needs of the population. Providing information on the changes in market performance over long periods sheds light on its determinants as well. However, to accomplish this, the metric systems for human resources must replace their individual, non-linked and cross-sectional indicators with linked and continuous indicators that can serve to analyze the multiple aspects of the labor market over time. Such indicators are of particular relevance for those who plan the workforce in the educational and health sectors, especially in countries that have access to systematic data from official sources and sociodemographic surveys.

Our study pursued a twofold objective: (1) to propose a performance metric for the nursing labor market and measure its magnitude; and (2) to analyze the temporal patterns of this market over the last fifteen years by socioeconomic region, with emphasis on employment differentials in the public and private sectors. In the next section, we present the conceptual elements that guided this study. We then describe our methods and define the proposed labor performance metric. Finally, we present the main results, discuss their implications for the Mexican health system and the health systems of other similar countries, and draw a set of conclusions.

## Conceptual framework

### The structure and equilibrium of markets

According to the classic perspective of markets, supply and demand of labor are balanced when the maximum number of individuals is engaged in the existing market spaces–whether public or private. However, supply and demand forces are dynamic, rendering it difficult to hold an equilibrium in markets. Unemployment, a classic indicator of disequilibrium, reflects an over-abundant labor force, or an excessive supply of labor, relative to demand. In professional markets, underemployment provides a key indicator as well. While the latter denotes low working hours and incomes in general labor markets, in professional markets, it is understood as the volume of professionals who find no work in their corresponding markets and end up working in areas unrelated to their professional expertise.

As mentioned above, precarious employment is a growing phenomenon among health professionals. This indicates a greater level of disequilibrium in the market since it deprives salaried workers of labor conditions adequate for fulfilling their tasks commensurate with their educational level [13]. Although the nature of precarious employment differs from that of unemployment and underemployment, it is important to understand how it contributes to market imbalance.

The proposed performance assessment is relevant in that it offers a comprehensive perspective of the labor market. Performance should not be understood merely as the capacity to balance the supply and demand of health workers. It should also be seen as the ability to provide workers with conditions that allow them to function as a key element for achieving the objectives of health systems and institutions. From the supply standpoint, these conditions involve the training of students at all levels; on the demand side, they concern the assurance of protected labor conditions, in accordance with international conventions and national regulations.

### The determinants of market deterioration

The deterioration of professional labor markets is subject to exogenous and endogenous structural determinants that interact with one another despite their different natures [14]. The first relates to a transformation in global economic relations as powers compete for capital reproduction spaces across the globe. This phenomenon has permeated all economic sectors, with recent emphasis on service production. It is in this particular context that health services and their associated endogenous determinants evolve. In low- and middle-income countries, the incorporation of capital has managed to insinuate itself into health systems of all types. To a varying degree, health system reforms facilitate the incorporation of private capital to finance the production of highly profitable services. This has modified labor relationships in the production of both private and public services as efforts have shifted their focus to the need for maximizing capital reproduction, minimizing costs and reducing the budgets of the public sector [15]. The effects of these exogenous determinants can be assessed by measuring the aforementioned indicators of unemployment, underemployment, and precarious employment, with the latter understood as a summary measure of specific job characteristics such as salary, benefits, excessive work and contractual instability.

### The structure of the nursing labor market in Mexico

Nursing professionals in Mexico are trained at three educational levels: (a) auxiliary, (b) technical and (c) university. The first two were initiated over a century ago in hospitals associated with religious orders and were later assumed by the government. Public and private hospitals have continued training nurses to ensure an ad hoc production of personnel that meets their specific needs. Social Security institutions provide exclusively technical training including technical specialization programs. Aspiring nurses began attending university five decades ago, with the number of graduates growing exponentially over the past 20 years. Those who earn a graduate degree can pursue postgraduate (master and doctorate) credits and clinical specialization [16]. Demand for nursing graduates is segmented by educational level and the type of institution requiring personnel. Public institutions follow various recruitment regimes. Some, including the Ministry of Health, have opened their doors to university graduates as their production has grown, and have modified their structure of job and salary codes somewhat to distinguish among nursing educational levels [17]. Others, such as the Social Security institutions, prefer technical personnel who scale through the labor code structure as they acquire higher training [18]. Although the latter have begun to engage a larger number of university professionals in recent years, they have yet to adjust their structure of job and salary codes to differentiate technical from university-level personnel. Private hospitals are also incorporating more university professionals; however, they lack a common structure of job and salary codes and tend to offer precarious employment conditions [19].

### Performance assessment of professional labor markets

The performance of professional labor markets is often assessed on the basis of individual indicators, primarily unemployment; underemployment is evaluated to a lesser degree. Both reflect the extent to which specific markets have the capacity to incorporate personnel with the corresponding level of training [20]. A third indicator, precarious employment, reflects not the incapacity of markets to integrate individuals in areas corresponding to their professions, but their incapacity to engage them in such areas under protected and stable labor conditions**. **Furthermore, inadequate labor conditions have also been associated to reduced quality of care and patient safety [21, 22]. The set of indicators used for assessing the performance of professional markets therefore needs to be integrated and tracked over time. At present, analyses of the health labor market use indicators such as salary, work schedule, geographic distribution and productivity [23]. Indicators of labor waste are not considered even though this factor prevents thousands of workers from participating in the markets for which they were trained. Precariousness is also ignored given its recent incorporation into analyses of this type [24].

## Methods

### Settings/design and study participants

We performed a repeated cross-sectional analysis of secondary data on the Mexican nursing labor market collected between 2005 and 2019. We drew the information from the National Survey of Occupation and Employment (ENOE by its initials in Spanish), which is a public, cross-sectional, probabilistic and retrospective population-based survey representative at the national and state levels (Mexico has 32 states), including rural/urban strata. It is the main data source used for generating official labor indicators and their individual and household socioeconomic and demographic correlates. This survey is administered quarterly to individuals 15 years or older residing in more than 100,000 households [25].

The ENOE is designed as a rotating panel with cycles of five visits, so that each quarter, twenty percent of the original sample is replaced. Trained interviewers carry out standardized, structured face-to-face interviews with key household respondents. All participants provide the requisite informed consent. In order to eliminate the quarterly seasonality of the analyzed data, capture the maximum levels of heterogeneity in the data as well as reduce the redundancy of observation units, all households visited for the first time in each quarter and each year were used to select economically active nursing professionals as defined by the INEGI Career/Profession Coding Catalogue (*n* = 21,854). After excluding observations with incomplete information on the variables of interest, our final sample included 19,311 nurses throughout the study period, equivalent to 4,816,930 at the national level. We looked for potential differences between our analytical sample and those excluded because of missing data–particularly as regards covariates that could be associated with our outcome variable–but found no significant differences.

This study did not involve human participants and the data for the analysis were requested and obtained from the surveys public repository hosted at the INEGI (see in https://www.inegi.org.mx/programas/enoe/15ymas/#Microdatos). Therefore, the approval for this secondary analysis was not obtained from any Ethics, Research or Biosecurity Committee.

### Measures

#### Performance of the nursing labor market

We analyzed four performance indicators for the nursing labor market [13, 26]:

- Unemployment (yes = 1/no = 0), denoting nurses who did not work for a payment although they were available and desired to work (or change jobs);
- Underemployment (yes = 1/no = 0), denoting nurses who were employed outside the health sector and who were available but preferred to work outside the health sector (or change jobs);
- Employment (yes = 1/no = 0), denoting nurses who were employed in the health sector and were available to work (or change jobs), and
- Non-precarious employment (yes = 1/no = 0), denoting nurses who self-reported having non-precarious jobs [13], in terms of three dimensions: (i) the economic, which considers the level of income received in exchange for labor force, using minimum wage as a reference; (ii) the regulations, which include the contract and duration of the working day, specifically, whether or not there is a written contract; and (iii) occupational safety, which includes the affiliation to social security and/or social benefits, measured by being receiving services from health institutions and having at least one social benefit. Based on this elements, and following our previous studies [13, 27], a non-weighted score of additive work precariousness was built based on the sum of five dichotomous variables (yes = 1/no = 0): (i) salary: equal to 1 if the individual reported up to two times the minimum wage; (ii) workday: equal to 1 if the individual works less than 34 h or more than 48 h (part-time or extended time); (iii) contract: equal to 1 if the individual reported not having a written contract; (iv) social benefits: equal to 1 if the individual did not comply with this condition; (v) social security: equal to 1 if the individual self-reported not having access to services from health institutions. All these variables were counted as zero if they did not comply with the condition. Thus, each nurse could have a value of zero to five. Non-precarious employment (score equal to zero) in the health sector (yes = 1/no = 0) was our main proxy for performance of the nursing labor market.

#### Covariates

In line with previous studies [13], we analyzed the following data on the sociodemographic, labor and residence characteristics at the individual level: sex (female = 1/male = 0), age (≤ 24, 25–54 and ≥ 55 years) at the time of the survey [28], marital status (married/free union and single or divorced/widowed), education (bachelor's or other university degree), labor sector (public or private), number of jobs (1 or ≥ 2); place of residence (rural, semi-urban, urban and metropolitan), and the socioeconomic region of residence according to the official definition in Mexico [29].

### Statistical analysis

All analyses were performed using the *svy* module of the Stata MP 15.1 statistical package, and all descriptive and multivariate analyses were based on survey weights to account for the complex survey design. First, we quantified the distribution of nursing professionals according to their labor status (unemployed, underemployed or employed in the health sector) during the last three federal government administrations and the first year of the current administration: Vicente Fox (last two years in office: 2005–2006); Felipe Calderon (2007–2009 and 2010–2012); Enrique Peña (2013–2015 and 2016–2018); and Andres Manuel López-Obrador (first year in office: 2019). We then described the sociodemographic, labor and contextual characteristics of the surveyed nurses according to their labor status and levels of employment precariousness, indicating the means and percentages of these variables with a 95% CI . In addition, we determined the distribution of employed nursing professionals according to their levels of employment precariousness, government period and labor sector.

We assessed the influence of the labor sector on the likelihood of having non-precarious work by estimating two groups of repeated cross-sectional, multiple logistic regression models [30–32]. The first group referred to the sample of underemployed nurses and other workers in the health sector, while the second pertained to the sample of health-sector employees. We constructed three models for each sample, differentiated by means of adjusted covariates: the first model was adjusted only by quarter (*Trim*) and survey year (*Survey*) as well as by the relationship between the labor sector (*Sector*) and survey year; the second was further adjusted by the individual characteristics mentioned above; and the third adjusted model (the most comprehensive) included the contextual features. The three models were specified as follows:

$${\text{Log}}\left( {\frac{p}{1 - p}} \right) = \beta_{0} + \beta_{{{\text{sector}}}} {\text{Sector}}_{j} + \beta_{{{\text{trim}}}} {\text{Trim}}_{j} + \beta_{{{\text{survey}}}} {\text{Survey}}_{j} + \mathop \sum \limits_{k} \beta_{k} Z_{kj} + \varepsilon_{j} ,$$

where *p* represented the occurrence probability of the event of interest, and *Z* a vector that included the sociodemographic, labor and residence characteristics mentioned above for labor sector *k*, measured for each nursing professional *j* surveyed. *β*′s and ${\varepsilon }_{j}$ represented a vector of parameters to be estimated and the error term, respectively. Post-estimate and goodness-of-fit tests showed that the regression models exhibiting a good fit for the data had been correctly specified. Lastly, we adjusted the prevalence rates and corresponding 95% CIs of non-precarious health-sector employees by survey period and socioeconomic region of residence.

## Results

### Descriptive results

**Fig. 1:**
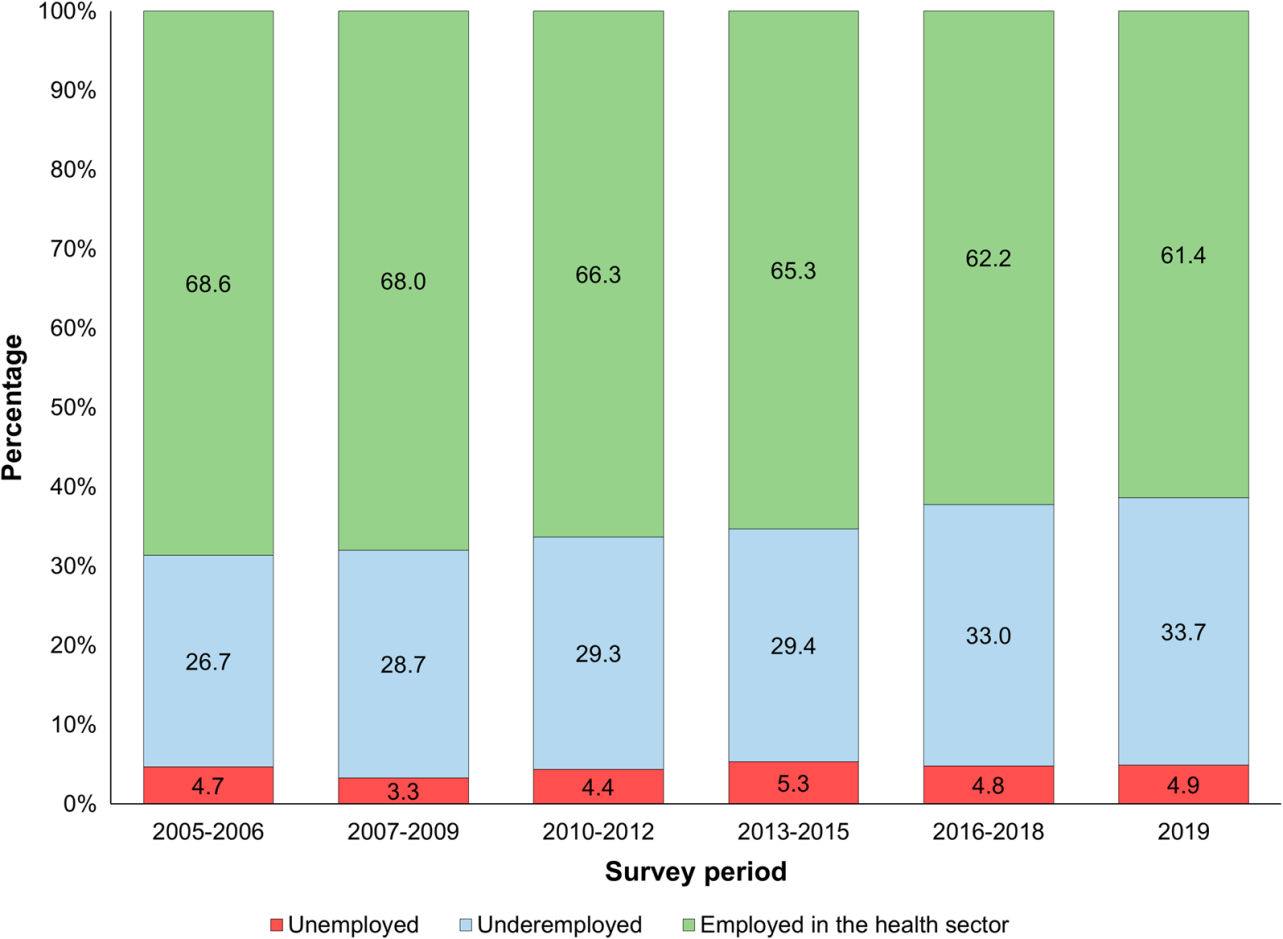
Distribution of nursing professionals according to labor condition. Mexico, 2005–2019. Estimates took into account the design effect of the survey. Data source: National Occupation and Employment Survey (ENOE in Spanish) 2005–2019

During the period analyzed, the unemployment rate among our sample of nursing professionals varied by 5%, the percentage of those employed in the health sector diminished by 10.5% (dropping from 68.6% in 2005–2006 to 61.4% in 2019), and the percentage of those underemployed grew by 26.2% (rising from 26.7% in 2005–2006 to 33.7% in 2019) (Fig. 1).

During the same period, 25.2% of the study population self-reported precarious underemployment and 4.9% non-precarious underemployment (Table 1). 28.7% of the sample self-reported non-precarious and 36.6% precarious employment conditions in the health sector. More men than women reported being precariously (29.7% vs. 24.5%) and not precariously (6.7% vs. 4.6%) underemployed. Conversely, a smaller percentage of men reported being employed in the public and private health sectors, whether precariously (25.0% vs. 29.5%) or not (31.9% vs. 37.3%). The unemployment rate decreased with the age of the nursing professionals; this was in contrast to what we observed with non-precarious underemployment, where the percentage of underemployed nurses increased with age.

**Table 1:** Main characteristics of the nursing professionals analyzed. Mexico, 2005–2019

	Unemployed	Underemployed		Employees in the health sector	
		Non-precarious	Precarious	Non-precarious	Precarious
Weighted sample	*219 652*	*235 548*	*1 214 348*	*1 762 996*	*1 384 386*
%	*4.6*	*4.9*	*25.2*	*36.6*	*28.7*
% and 95% CI					
Sociodemographic and labor characteristics					
Men	6.7 (4.9–8.5)	6.7 (5.2–8.2)	29.7 (26.8–32.6)	31.9 (28.9–34.8)	25.0 (22.4–27.6)
Women	4.2 (3.7–4.7)	4.6 (4.1–5.1)	24.5 (23.4–25.7)	37.3 (36.1–38.6)	29.3 (28.1–30.5)
Age (y)					
≤ 24	11.4 (9.6–13.3)	3.9 (2.8–5.0)	34.7 (32.1–37.4)	15.1 (12.8–17.4)	34.8 (32.2–37.4)
25–54	3.3 (2.8–3.8)	5.0 (4.5–5.5)	21.9 (20.7–23.0)	41.9 (40.5–43.3)	28.0 (26.7–29.2)
≥ 55	1.3 (0.5–2.2)	6.0 (3.9–8.0)	40.6 (36.1–45.1)	30.7 (26.5–34.9)	21.4 (17.8–25.1)
Marital status					
Married or free union	2.8 (2.3–3.3)	5.2 (4.5–5.8)	24.2 (22.8–25.5)	40.6 (39.1–42.2)	27.2 (25.8–28.7)
Single	7.8 (6.7–9.0)	4.2 (3.4–5.0)	25.4 (23.6–27.3)	30.4 (28.5–32.3)	32.1 (30.2–34.0)
Divorced or widowed	2.8 (1.8–3.9)	5.6 (4.3–6.9)	29.6 (26.3–32.8)	36.3 (33.1–39.6)	25.6 (22.7–28.6)
University education					
No	4.7 (3.9–5.5)	5.5 (4.8–6.2)	29.9 (28.2–31.6)	32.7 (31.0–34.4)	27.2 (25.6–28.8)
Yes	4.4 (3.8–5.1)	4.3 (3.7–4.9)	20.8 (19.5–22.0)	40.3 (38.7–41.9)	30.2 (28.7–31.6)
Labor sector					
Private	–	7.0 (6.2–7.9)	57.9 (56.0–59.8)	13.8 (12.5–15.1)	21.2 (19.7–22.8)
Public	–	3.8 (3.3–4.4)	5.0 (4.3–5.6)	55.1 (53.5–56.6)	36.2 (34.6–37.7)
No. of jobs					
One	–	4.8 (4.3–5.3)	24.9 (23.8–26.0)	37.0 (35.8–38.3)	28.5 (27.3–29.6)
Two or more	–	6.1 (3.7–8.5)	30.3 (25.9–34.6)	30.1 (26.2–34.0)	33.5 (29.4–37.6)
Place of residence					
Rural	4.2 (2.7–5.7)	3.7 (2.0–5.4)	31.2 (27.1–35.3)	30.8 (27.0–34.6)	30.1 (26.4–33.7)
Semi-urban (peri-urban)	4.8 (3.4–6.2)	3.3 (2.2–4.4)	24.7 (21.7–27.7)	34.6 (30.9–38.3)	32.6 (29.2–36.0)
Urban	4.9 (3.6–6.2)	3.4 (2.4–4.4)	27.3 (24.4–30.2)	35.3 (32.1–38.4)	29.1 (26.2–32.0)
Metropolitan	4.5 (3.8–5.1)	5.7 (5.1–6.3)	24.0 (22.7–25.3)	38.0 (36.6–39.5)	27.8 (26.5–29.1)
Socioeconomic region					
Poorest	4.1 (2.8–5.3)	3.6 (2.5–4.8)	24.8 (21.9–27.6)	33.6 (30.7–36.6)	33.9 (31.0–36.8)
2	4.0 (3.0–5.0)	3.6 (2.5–4.6)	26.7 (24.2–29.3)	35.4 (32.7–38.1)	30.3 (27.8–32.8)
3	3.8 (2.6–4.9)	4.5 (3.1–5.9)	23.8 (20.6–26.9)	36.8 (33.5–40.0)	31.2 (28.0–34.5)
4	4.7 (3.4–6.0)	3.7 (2.8–4.6)	26.3 (23.8–28.9)	35.3 (32.5–38.0)	30.0 (27.3–32.7)
5	4.3 (3.1–5.4)	7.3 (5.8–8.7)	24.2 (22.1–26.4)	38.8 (36.1–41.4)	25.5 (23.2–27.7)
Wealthiest	5.4 (4.4–6.4)	6.2 (5.2–7.2)	24.3 (22.2–26.5)	38.4 (36.0–40.8)	25.7 (23.8–27.7)

Estimates considered the design effect of the survey. Data source: National Occupation and Employment Survey (ENOE in Spanish) 2005–2019

In addition, we observed a U-inverse relationship between the percentage of nurses employed in the health sector under non-precarious conditions and age: 15.1% (95% CI: 12.8%-17.4%) among individuals 24 years old or younger, 41.9% (95% CI: 40.5%-43.3%) in those 25 to 54 years old, and 30.7% (95% CI: 26.5%-39.6%) in those over 54 years old. Surprisingly, the percentage of unemployment was similar for nurses who had received a university education (4.4%, 95% CI: 3.8%-5.1%) and those who reported having a technical background (4.7%, 95% CI: 3.9%-5.5%). This was not the case regarding the percentage of people in the other job categories. In particular, the percentage of nurses employed in the health sector under non-precarious conditions was 23% higher (32.7% vs. 40.3%) for those with university-level education. Precarious underemployment was more prevalent in the private (57.9%) than in the public (5.0%) sector. However, the percentage of nursing professionals employed under precarious and non-precarious conditions was higher among those employed in the public (36.2%, 95% CI: 34.6%-37.7% and 55.1%, 95% CI: 53.5%-56.6%) as opposed to the private (21.2%, 95% CI: 19.7%-22.8% and 13.8%, 95% CI: 12.5%-15.1%) sector, respectively. The prevalence rates of both non-precarious underemployment and non-precarious employment in the health sector increased according to the degree of urbanity of the place of residence and the level of wealth of the region in which the nursing professionals resided (Table 1).

**Fig. 2:**
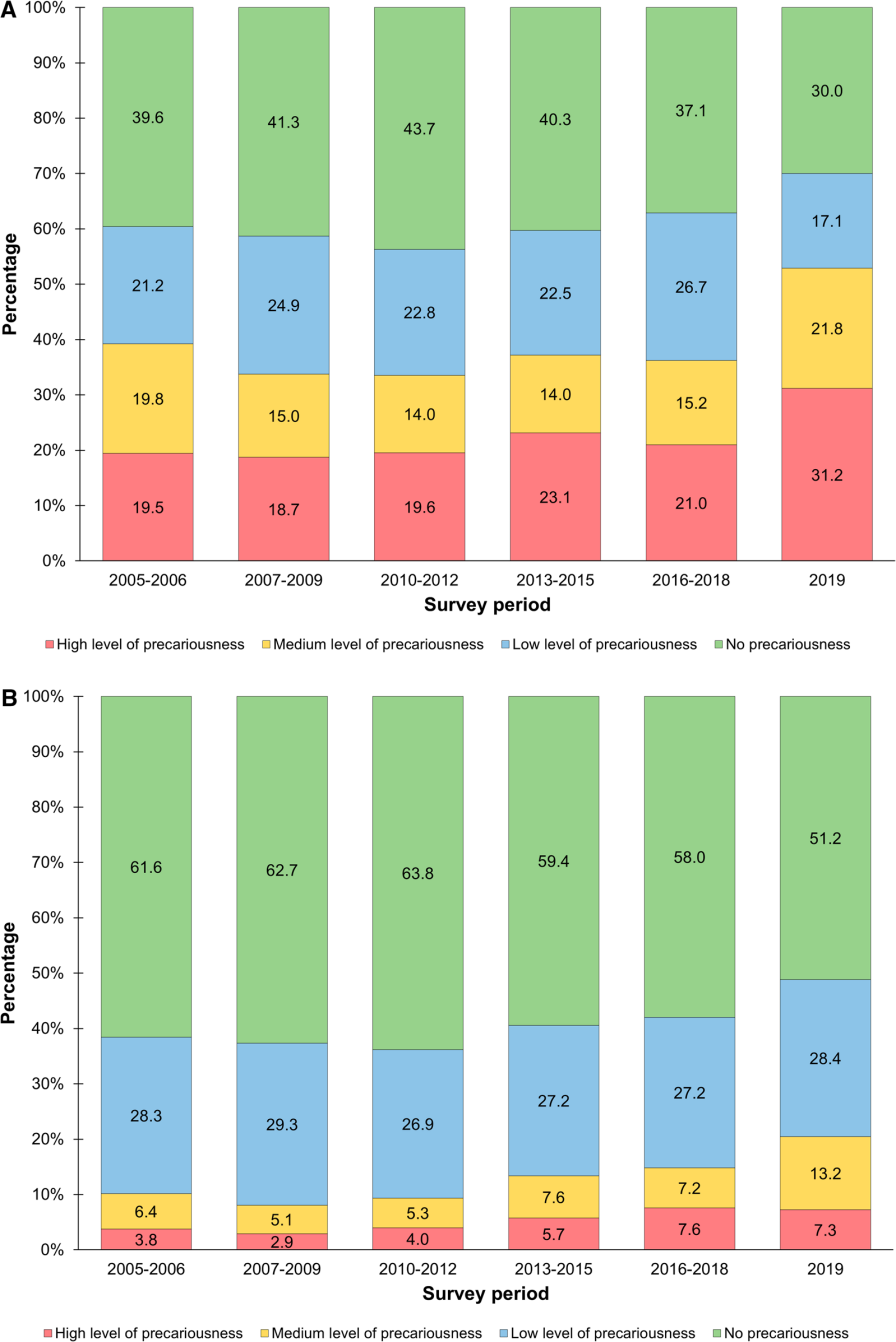
Distribution of nursing professionals according to level of employment precariousness and labor sector. Mexico, 2005–2019. **A** Employees in the private sector. **B** Employees in the public sector. Estimates considered the design effect of the survey. Data source: National Occupation and Employment Survey (ENOE in Spanish) 2005–2019

We also observed that the percentage of employees in the private sector working under highly precarious conditions had grown notably over the last fifteen years, from 19.5% in 2005–2006 to 31.2% in 2019 (Fig. 2a). On the other hand, the percentage of employees working in non- or low-precarious conditions in the private sector decreased by 22.5% (from 60.8% in 2005–2006 to 47.1% in 2019, *P* < 0.001). The situation was different in the public sphere (Fig. 2b). Although the percentage of nurses employed in highly precarious conditions practically doubled (from 3.8% in 2005–2006 to 7.3% in 2019), the percentage of those employed in non- or low-precarious conditions fell by 11.5% (from 89.9% in 2005–2006 to 79.6% in 2019).

### Regression results

**Fig. 3:**
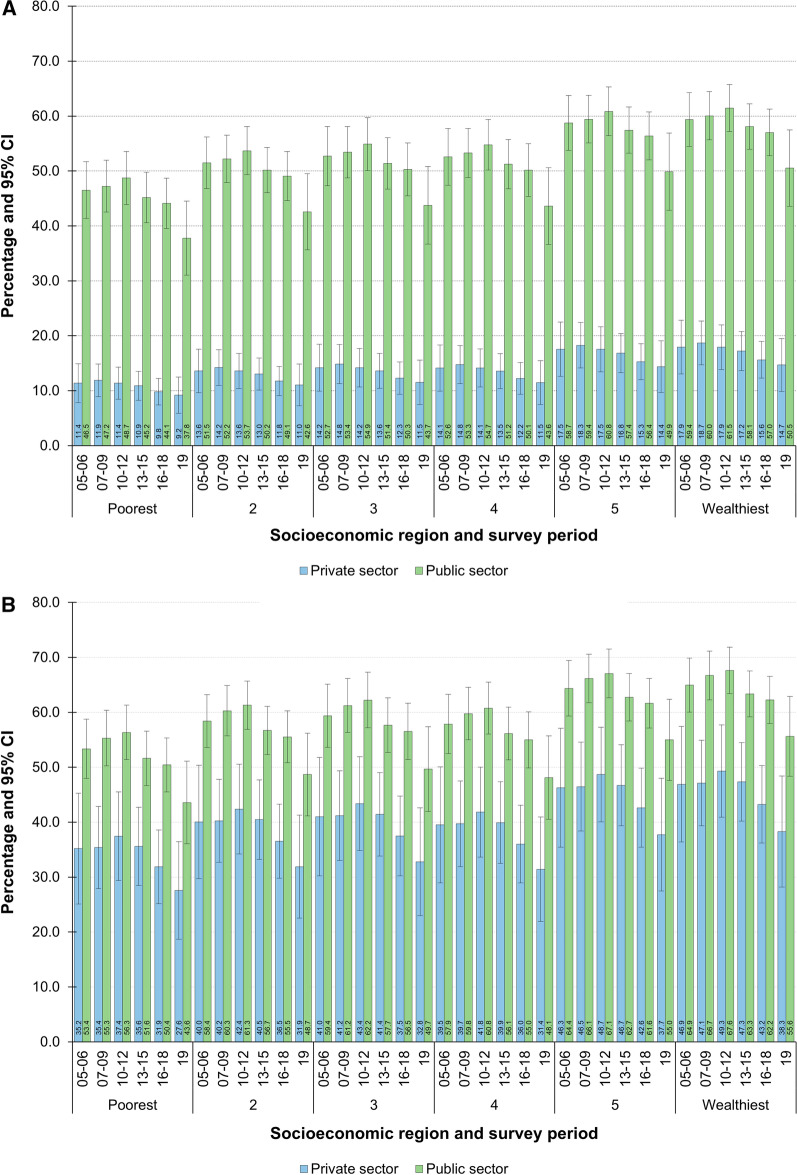
Prevalence of non-precarious employment according to labor sector and socioeconomic region. Mexico, 2005–2019. **A** Among underemployed and employed nurses in the health sector. **B** Among employed nurses in the health sector. Adjusted percentages after estimating model 3 in Table 2. Estimates took into account the design effect of the survey. Data source: National Occupation and Employment Survey (ENOE in Spanish) 2005–2019

The aOR of having a non-precarious job for nursing professionals who were underemployed or employed in the health sector, were higher among public-sector workers (aOR 7.11, 95% CI: 4.91–10.31). These odds ratios dropped considerably when the estimates were limited to those who reported being employed (aOR 2.17, 95% CI: 1.36–3.46) (Table 2). Among both female and male working nurses, the percentage of those employed in non-precarious conditions was notably higher in the public than in the private sector and increased according to the levels of wealth of their regions of residence, although this difference has been narrowing in recent years (Fig. 3a). Correspondingly, the percentage of people employed under non-precarious conditions in the health sector grew according to the level of regional wealth (Fig. 3b). These models showed that although this percentage had been declining in the public sector, it tended to equal that estimated for the private sector, especially at the end of the period analyzed.

**Table 2:** Regression analysis results for labor sector influence on non-precarious employment. Mexico, 2005–2019

	Underemployed and employed in the health sector			Employed in health sector		
	Model 1	Model 2	Model 3	Model 1	Model 2	Model 3
aOR's and 95% CI						
Labor sector						
Private	Ref	Ref	Ref	Ref	Ref	Ref
Public	8.04***	6.78***	7.11***	2.61***	2.03**	2.17**
	**(7.09–9.12)**	**(4.68–9.82)**	**(4.91–10.31)**	**(1.69–4.02)**	**(1.27–3.23)**	**(1.36–3.46)**
Adjusted covariates						
Survey quarter	*Yes*	*Yes*	*Yes*	*Yes*	*Yes*	*Yes*
Survey year	*Yes*	*Yes*	*Yes*	*Yes*	*Yes*	*Yes*
Labor sector × survey year	*Yes*	*Yes*	*Yes*	*Yes*	*Yes*	*Yes*
Sociodemographics and labor characteristics	*No*	*Yes*	*Yes*	*No*	*Yes*	*Yes*
Place of residence characteristics	*No*	*No*	*Yes*	*No*	*No*	*Yes*
Observations	18,560	18,560	18,560	12,924	12,924	12,924
Goodness-of-fit test (Hosmer and Lemeshow)						
*F*-adjusted test statistic	1.11	2.05	0.98	0.83	0.58	0.74
*P* > F	0.36	0.03	0.45	0.59	0.82	0.67

Estimates considered the design effect of the survey

*Data source* National Occupation and Employment Survey (ENOE in Spanish) 2005–2019

***P < 0.001, **P < 0.01, *P < 0.05

## Discussion

In this paper, we propose a composite metric for assessing the performance of the Mexican labor market for nursing professionals and describe our analysis of its predictors and temporal patterns. Our strategy recognizes the complex nature of this market, manifested in a number of characteristics. First, the nursing market in Mexico, as in many other LMICs, engages nurses with very dissimilar levels of training ranging from nine to twenty years of formal education [33]. Second, to date, the labor market has been unable to generate differences in salaries commensurate with the level of training. For example, it has been documented that nurses with 16 years of training often earn incomes similar to those of individuals with less education [34]. Third, in the last 10 years, the market has failed to provide adequate employment opportunities corresponding to the increased availability of highly trained nurses [35, 36]. Fourth, the unprecedented increase in the demand for nursing personnel by private institutions in the last decade reflects the growth of this sector in the Mexican health system [27].

The performance indicators analyzed in our study raised a set of specific elements to consider. Two of them, unemployment and underemployment, establish the capacity of the markets to absorb available professionals. The behavior of the former remained stable at approximately 5% during the period studied, suggesting low sensitivity to variations in the nursing labor market. Underemployment, on the other hand, grew noticeably (26%) over this period, implying that graduate nurses are increasingly seeking opportunities in other markets outside of nursing. The combined effect of both indicators revealed that, by 2019, only 61% of nursing professionals were employed as nurses. The third indicator, precarious employment*,* showed the weakness of the market in offering working conditions in accordance with the legislation in force in Mexico [37]. The precariousness of the nursing labor market is a phenomenon that not only grew significantly during the period studied, but also became increasingly widespread throughout the country [13].

These indicators offer a better overview of the complex nursing market when analyzed together [38]. Thus, we found that, of the total number of underemployed nurses of both sexes, the vast majority worked in precarious conditions. This reflected the fact that their employment status did not necessarily improve when they were forced to seek work in labor markets outside their own profession. Some sociodemographic characteristics also established differences. The men in our sample faced a higher risk of underemployment and precarious employment; this fact was linked to the increase in the proportion of men in the labor market observed in recent years, at a time when the number of positions in health institutions has declined and working conditions have deteriorated. Age also established differences. As happens in many professional and non-professional markets, the intermediate ages in our sample were those where the best working conditions were concentrated; older individuals tended to be more underemployed once they left the professional market. On the other hand, as reported in other countries, nursing professionals with university training observed an increase in the labor market [39], particularly in non-precarious positions. Therefore, it is possible that the market is preferring to recruit nurses with bachelor's degrees given that, as previously noted, the salary differences among educational levels are very small.

The results emphasize the restructuring of the nursing labor market in Mexico, with significant growth in the private sector [27]. This labor restructuring was preceded by a restructuring of the health-care market as a whole. While 52% of health spending in the country goes to the private sector and the remaining 48% to the public sector, the latter provides a greater volume of services (60%). The growth of the private sector has clearly attracted nurses who find no opportunities in the public sector, yet private sector demand includes a high proportion of precarious jobs. It should also be noted that, although there are differences between the two sectors, the precarious nature of intermediate- and high-level positions in the public sector has increased in recent years as well. It is feasible to assume that in the coming years the differences between the two sectors could be reduced to a minimum if this trend continues.

The nursing labor market has also undergone other changes in recent years, particularly related to differences between job categories and between the public and private sectors. Until 2000, hospital-trained technical nurses dominated the market. This training was carried out in health units and institutions to meet their own demand. The most important case was the Mexican Social Security Institute, which for more than 40 years focused its demand exclusively on recruiting technical nurses and undertake their specialization through post-technical courses offered by the institution itself [40]. The growth in the supply of nurses trained in universities has been taken advantage of by the Ministry of Health and it has invested in their training as specialists and in postgraduate degrees (master's and doctoral). In the master's level training of advanced practice nurses in Mexico, only university-trained nurses are recruited and if this market expands in the coming years, technical nurses could lose spaces that are currently still protected. Other job niches have also opened up in the non-institutional private sector. A relevant case is home care. Through companies that contract their services or on an individual basis, the increase of nurses caring for terminal or chronic patients in their homes has been documented [41].

The participation of professional associations in the way in which these personnel are linked to the labor market has been scarce, as well as the participation in the generation of norms that regulate these forms of linkage. Recently, the major focus of these associations is to create certification guidelines for personnel, leaving as a secondary issue the defense of the labor rights of their members [42].

Regression analyses for underemployment showed that, in labor markets unconnected to health, public sector positions are up to eight times more likely to offer non-precarious conditions compared with private employment. This difference was radically reduced in the professional nursing market, suggesting that the protective factor of employment in the public sector is not very different from that in the private sector [43]. These slight differences could stem from the fact that, over the past 20 years, the public sector began to generate demand for nurses (and other health professionals) based on temporary contracts with no guaranteed labor rights. Although efforts were made to improve temporary contract conditions [44], they were insufficient for reducing the level of precariousness.

It is important to note the geographic differences in the professional nursing market. Mexico has made significant efforts to provide health care to poor populations that are generally located in rural and peri-urban areas. From the perspective of the nursing labor market, we observed that precarious employment is much more prevalent in small and medium-sized locations. Therefore, when we talk about health-service markets, it is important to consider the care for the poorest populations on which government policy in Mexico has focused in the last 30 years. Wealthier (mostly urban) areas of the country may offer more protected and stable employment and therefore attract a higher volume of nurses. However, the decline in both public and private protected employment shows that the deterioration of the nursing labor market has increased in the poorest areas of the country. From this perspective, bringing nurses to poor, rural and peri-urban areas requires the development of a predefined policy where the government establishes investment priorities not only to guarantee the public access to services, but also protected jobs.

In summary, an analysis of professional labor markets requires the use of various types of articulated indicators to account for the complex phenomena that characterize these markets. In the case of the Mexican nursing market, we used three combined indicators: *unemployment, underemployment* and *precarious employment*. We adopted *unemployment* because it is the most common indicator used to analyze the performance of general labor markets. However, as we have shown, it has limited applicability in the case of professional markets. Underemployment, on the other hand, offers a variety of options for analyzing the incapacity of professional labor markets to incorporate graduates from professional schools as well as the risks this implies for falling into precarious working conditions. Precariousness exposes the risks that professional labor markets are facing as a result of globalization, including the reduction of the protective effect of public institutions.

Our study had several limitations. First, although the ENOE is a high-quality, systemic and periodic population-based survey, ours was a cross-sectional study and thus provided information only on associations, not causal effects. Second, the study used self-reported measurements; therefore, the results may have been affected by recall bias. They may also have been affected by inaccurate weighting of the analyzed performance dimensions. To validate the proposed metric, future studies in Mexico and other LMICs should consider different approaches such as consulting labor-market experts (i.e., using Delphi methods) and comparing results with those of countries with similar data sources. Third, the proposed metric did not incorporate the duration or chronicity of unemployment, underemployment and precarious employment. The incorporation of this metric requires a longitudinal structure (prospective or retrospective) of the analyzed data. Future analyses should address this dimension of labor market performance. On the other hand, our study had several strengths. The three dimensions of the proposed metric allowed for a highly comprehensive study of labor market performance. They covered the quantity and quality of demand/supply of employment in the nursing sector. In addition, the data used encompassed a 14-year period, enhancing the reliability of our results.

In the current context, strategies for evaluating the performance of the labor market for health workers must consider the changes that the COVID-19 pandemic is imposing around the world. Dealing with the pandemic has required hiring thousands of workers under a variety of conditions. In Mexico, the government has engaged approximately 45,000 health workers, primarily nurses and doctors, on a temporary basis during the acute phase of the epidemic. The majority of these workers have been contracted to care for COVID-19 patients in hospital settings, with very few assigned to work in the community. Judging by the high number of infected employees, the working conditions for newly hired staff are often inadequate. Infected workers on short-term contracts are sent to quarantine and their contracts generally terminated. The COVID-19 pandemic has exposed the stark vulnerability of those in precarious employment conditions; this has not undermined the ability of these health workers to adequately perform their duties. It is crucial to provide labor protection to newly hired workers if the health system is to achieve its goal of improving the health of the population.

Finally, from the discussion some basic policy recommendations emerge. First, it is important to implement a planning process for the training of nurses in the country, including both public and private schools to calibrate the production according to future health systems requirements. Second, a re-training program is needed to update underemployed nurses’ knowledge to seek employment opportunities in health institutions and capitalize the social investment made in their original training. Third, to pass and enforce legislation to combat labor market practices that attempt against labor rights including tertiarization, temporary contracting, unfair salaries, among others. Fourth, to implement an encompassing regulatory mechanism to promote high-quality training at schools, fair recruitment mechanisms and appropriate labor conditions in public and private health units. Despite globalization of training and labor relations is expanding, not all recommendations could be relevant to the reality of other LMIC and industrialized ones. In any case, they require analysis and adaptation.

## Data Availability

The dataset analyzed during this study is available from the corresponding author on request.

## Abbreviations

ILO: International Labor Office
ENOE: National Survey of Occupation and Employment
INEGI: National Institute of Statistics and Geography
CI: Confidence interval
aOR: Adjusted odds ratio
LMICs: Low- and middle-income countries
COVID-19: Coronavirus disease

## Declarations

### Study registration

Does not apply.
